# Epigenetics: Mechanisms, potential roles, and therapeutic strategies in cancer progression

**DOI:** 10.1016/j.gendis.2023.04.040

**Published:** 2023-07-06

**Authors:** Dong Wang, Yan Zhang, Qingbo Li, Yu Li, Wen Li, Ao Zhang, Jingxuan Xu, Jingyan Meng, Lin Tang, Shuhua Lyu

**Affiliations:** a Graduate School, Tianjin University of Traditional Chinese Medicine, Tianjin 301617, China; b College of Traditional Chinese Medicine, Tianjin University of Traditional Chinese Medicine, Tianjin 301617, China; c Department of Pathology, Tianjin Union Medical Center, Tianjin 300121, China

**Keywords:** Cancer, Epigenetics, Gene expression, Heterogeneity, Tumor microenvironment

## Abstract

Mutations or abnormal expression of oncogenes and tumor suppressor genes are known to cause cancer. Recent studies have shown that epigenetic modifications are key drivers of cancer development and progression. Nevertheless, the mechanistic role of epigenetic dysregulation in the tumor microenvironment is not fully understood. Here, we reviewed the role of epigenetic modifications of cancer cells and non-cancer cells in the tumor microenvironment and recent research advances in cancer epigenetic drugs. In addition, we discussed the great potential of epigenetic combination therapies in the clinical treatment of cancer. However, there are still some challenges in the field of cancer epigenetics, such as epigenetic tumor heterogeneity, epigenetic drug heterogeneity, and crosstalk between epigenetics, proteomics, metabolomics, and other omics, which may be the focus and difficulty of cancer treatment in the future. In conclusion, epigenetic modifications in the tumor microenvironment are essential for future epigenetic drug development and the comprehensive treatment of cancer. Epigenetic combination therapy may be a novel strategy for the future clinical treatment of cancer.

## Introduction

In February 2022, the National Cancer Center of China released the latest cancer report. In 2016, approximately 4.064 million new cancer cases and 2.413 million new cancer deaths were reported in China. The overall incidence and mortality of cancer in China are still on the rise.^1^ At the moment, the comprehensive treatment of cancer, such as surgery, radiotherapy, chemotherapy, targeted therapy, immunotherapy, traditional Chinese medicine (TCM), and combination therapy, can slow down the progression of cancer to some extent, but the recurrence and drug resistance severely affect the quality of life and prognosis of patients.^2^ As a result, active research and development of new treatments and drugs have significant clinical implications for cancer patients' overall survival and prognosis.

With the advancement of epigenetics in recent decades, epigenetic modification is now thought to play a role in the onset and progression of cancer.^3^ Epigenetic modifications in cancer mainly include DNA methylation, histone modification, and non-coding RNA (ncRNA) mutation or abnormal expression, which may lead to the occurrence and progression of cancer.^4^ Thus, research on epigenetic alterations in cancer cells has reshaped the way we treat cancer and guided the development of novel anticancer treatments and drugs that target epigenetic factors.

As new cancer cases and mortality rates increase worldwide, there is a greater need for effective biomarkers for early screening, diagnosis, and prognostic testing.^5^ Recent research has found that epigenetic alterations, such as abnormal promoter methylation, altered histone modifications, and ncRNA, play an important role in the development of cancer.^6^ In addition, with thorough research and the application of high-throughput technology, the impact of other epigenetic modifications on various cancers has also been confirmed.^7^ Therefore, epigenetic alterations could be used as potential biomarkers for early cancer detection and diagnosis.

With the application of technologies such as single-cell genomics and epigenetics, abnormal expression or mutation of genes induced by epigenetic alterations have been identified and proved to be drivers of cancer.^5^^,^^8^ At the same time, the research and development of drugs for cancer epigenetic modification are in full swing. Currently, epigenetic drugs approved for clinical use by the Food and Drug Administration (FDA) are primarily DNA methyltransferase (DNMT) inhibitors and histone deacetylase (HDAC) inhibitors, such as azacitidine, decitabine, enasidenib, vorinostat, and panobinostat.^9^ Although epigenetic drugs have achieved certain effects in the clinical treatment of cancer, there are still challenges such as the limited clinical efficacy of monotherapy and cancer epigenetic heterogeneity.^10^ In summary, epigenetic drugs combined with chemical drugs, targeted drugs, immune drugs, TCM, or a multi-drug combination strategy may be the focus of future cancer treatment research. Epigenetic combination therapy may be a promising strategy for the clinical treatment of cancer.

## Epigenetic modifications play an essential role in cancer progression

Epigenetic modification, such as DNA methylation, histone modification, and ncRNA primarily regulates gene expression and does not affect the base sequence of DNA itself. Dysfunction of epigenetic genomes has been linked to abnormal transcriptional expression, which promotes the occurrence and progression of cancer. Therefore, regulating epigenetic modification has important clinical implications for the clinical treatment, diagnosis, and prognosis of cancer.

## DNA methylation

**Figure 1:**
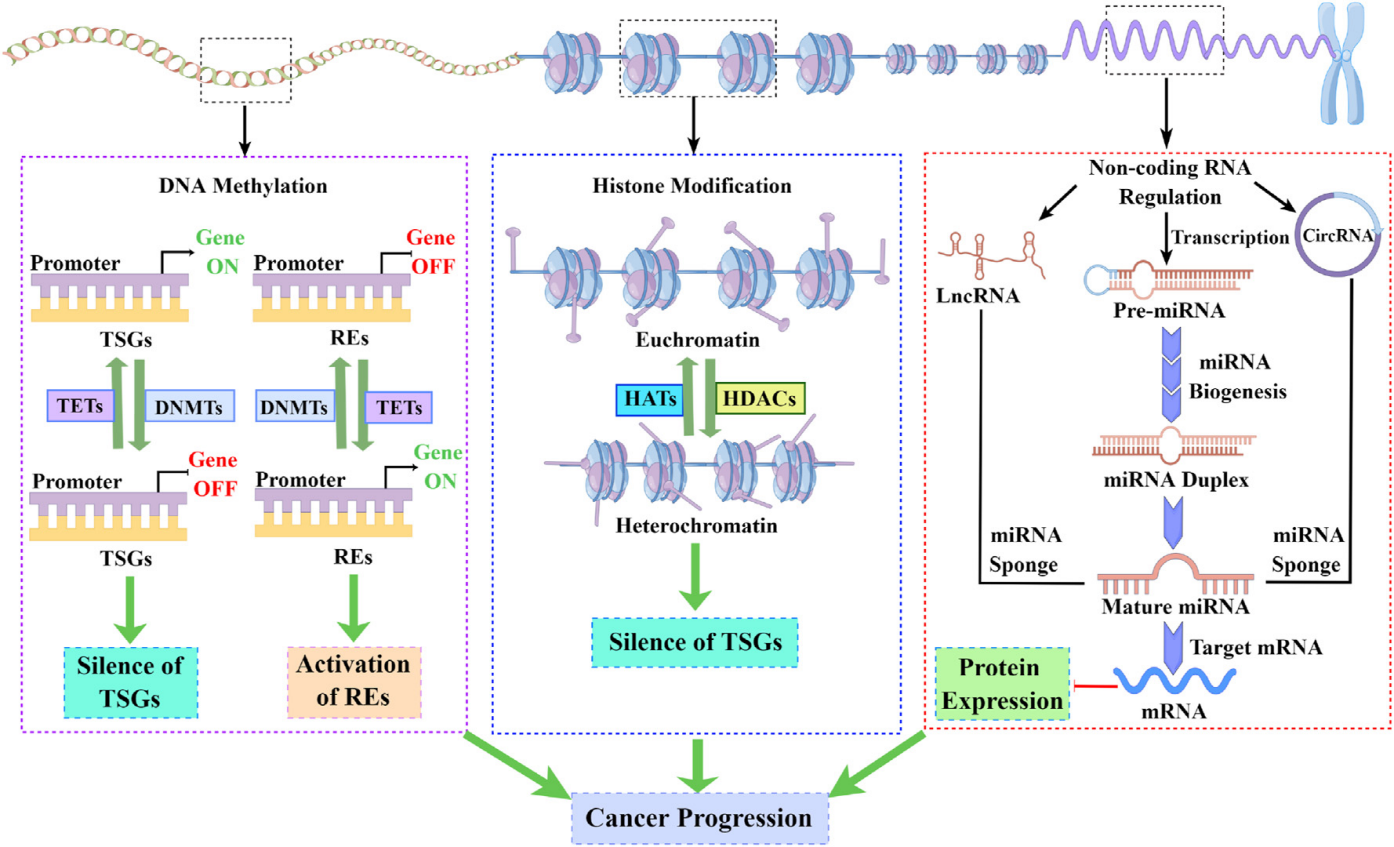
The role of epigenetic alterations in cancer progression. Epigenetic dysregulation of DNA methylation, histone modifications, and non-coding RNAs induces the silencing of tumor suppressor genes (TSGs) and activation of repetitive elements (REs). In cancer, DNA methyltransferases (DNMTs) transfer methyl to cytosines in cytosine-phosphate-guanine islands in the promoter, inducing TSG hypermethylation. This promoter hypermethylation leads to transcriptional repression of TSGs and promotes cancer progression. Conversely, eleven-translocation (TET) enzyme-induced promoter demethylation transcriptionally activates REs and leads to genomic instability and cancer progression. In histone modifications, histone deacetylases (HDACs) convert euchromatin to heterochromatin by removing acetyl groups from histone tails, leading to the silencing of TSGs and chromatin. Histone acetyltransferases (HATs), on the other hand, reverse this process, controlling histone acetylation levels and cancer progression. As a result, aberrant non-coding RNA regulation, particularly miRNA, targets mRNA and suppresses its protein expression. In addition, lncRNA and circRNA can act as miRNA sponges and regulate the expression of miRNAs, mRNAs, and their proteins. Taken together, aberrant epigenetic modifications promote cancer progression.

DNA methylation is one of the most extensively researched epigenetic modifications. DNA methylation can ensure proper regulation of gene expression and stable gene silencing.^11^ DNA methylation is primarily mediated by DNMT1, DNMT3a, and DNMT3b, which transfer the methyl group provided by S-adenosylmethionine to the carbon atom at the 5-position of cytosine to form 5-methylcytosine.^12^ It is well known that DNMT is a group of conserved DNA modification enzymes responsible for the establishment and maintenance of methylation patterns. DNMT1 mainly targets semi-methylated DNA and transmits parental DNA methylation information to offspring DNA after DNA replication. DNMT 3a and DNMT 3b are responsible for regulating *de novo* methylation, and S-adenosylmethionine is used as a methyl donor to methylate unmethylated cytosine-phosphate-guanine sites.^13^ DNA methylation or demethylation of gene promoter and enhancer sites has been linked to the occurrence and progression of cancer.^14^ Enhancer sequence mutations, changes in enhancer-promoter communication, epigenetic enzyme dysregulation, and binding of enhancer transcription factors lead to enhancer dysfunction, which promotes the transcriptional progression of cancer.^15^ Furthermore, abnormal methylation of the promoter may lead to the silencing of tumor suppressor genes, affecting their related transcription pathways and leading to cancer progression, as described in Figure 1.^16^ However, unlike genetic alterations, DNA methylation is reversible, making it a promising therapeutic strategy.^17^ Notably, DNA methylation can also be applied to the diagnosis of a variety of clinical diseases. Almost all tissues and body fluids can be used for DNA methylation analysis, including paraffin tissue, sputum, saliva, urine, even fetal membranes, umbilical cords, and placenta. In summary, DNA methylation can be applied to the early diagnosis of cancer, and the development of DNA methylation-based drugs plays an important role in the clinical treatment of cancer.

## Histone modification

Histone modification affects not only gene transcription, DNA replication, and DNA repair but also chromatin status.^18^ Histone acetylation is essential for gene transcription. It can reduce the electrostatic interaction between negatively charged DNA and histones by neutralizing the basic charge on unmodified lysine residues, thereby affecting the chromatin state. Histone acetyltransferase and HDAC are the key enzymes of histone acetylation, which dynamically regulate the level of histone acetylation, thereby changing gene expression, as shown in Figure 1.^19^ HDAC6 has been found to be overexpressed in a variety of malignant tumors, including acute myeloid leukemia (AML), colon cancer, and breast cancer. In addition, overexpression of HDAC6 is associated with advanced tumor stage and increased tumor invasiveness, with a lower survival period.^20^ In contrast to acetylation, histone methylation promotes the recruitment of regulatory factors, which eventually leads to gene inhibition. Histone methyltransferase-mediated histone methylation is a specific transcriptional regulator in response to cancer cell signals.^21^ Unlike other histone modifications, lysine 4 of histone 3 (H3K4), H3K36, and H3K79 methylation is associated with marker-activated transcription, whereas H3K9, H3K27, and H4K20 methylation is considered related to the silent chromatin state. These histone lysine methylations also interact with other histone modifications and DNA methylations to precisely regulate the expression of oncogenes or tumor suppressor genes.^22^ The enhancer of zeste homolog 2 (EZH2) has been shown to mediate trivalent methylation of H3K27 (H3K27me3), resulting in transcriptional repression of tumor suppressor genes.^23^ In addition, lysine-specific demethylase 1 (LSD1), which is overexpressed in non-small cell lung cancer, can target H3K4me2/me1 and H3K9me2/me1.^24^ It is worth noting that histone phosphorylation and ubiquitination are both important processes of histone modification. Histone phosphorylation is primarily the phosphorylation of specific amino acid residues by protein kinases by adding phosphate groups. Histone phosphorylation changes the structural conformation of proteins, causing cancer-target proteins to be activated or inactivated.^25^ Ubiquitin is a small (8.5 kDa) regulatory protein that is ubiquitinated onto the lysine residue of the substrate protein by ubiquitination. Histones, particularly H2A and H2B, are well-known ubiquitination substrates. Histone ubiquitination is essential for gene transcription, chromatin structure maintenance, and DNA repair.^26^ In conclusion, histone acetylation, methylation, phosphorylation, ubiquitination, and other epigenetic modifications affect the regulation of gene transcription, DNA replication, and DNA repair, all of which influence the occurrence and development of cancer. It is a promising target and biomarker for the clinical treatment of cancer.

## Regulation of non-coding RNA

Recent research has shown that, in addition to DNA methylation and histone modification, other epigenetic disorders also contribute to the occurrence and progression of cancer. ncRNA, which includes microRNA (miRNA), circular RNA (circRNA), and long non-coding RNA (lncRNA), does not encode proteins but is involved in regulating a variety of biological processes in cancer progression, such as proliferation, apoptosis, migration, and invasion.^27^ miRNA is a small RNA (about 22 nucleotides) that binds to complementary sequences in targeted mRNA and causes RNA-induced silencing complexes to silence or degrade targeted mRNA. Abnormally high expression of miR-126, miR-155, and miR-215 in breast cancer, colorectal cancer, and glioma promotes cancer progression. In addition, lncRNA and circRNA regulate gene expression via a variety of mechanisms. On the one hand, they can serve as miRNA baits to stop the degradation of specific mRNA. On the other hand, they can regulate the binding of transcription factors to promoters, thereby regulating the expression of targeted genes, as described in Figure 1. According to studies, HOTTIP is abnormally elevated in AML and regulates hematopoietic gene-related chromatin characteristics and transcription.^28^ Additionally, the TCF gene locus is the source of the lncRNA lnc-TCF7. Lnc-TCF7, which encourages liver cancer stem cells to self-renew, is significantly expressed in these cells.^29^ Studies have shown that the low expression of circ-CDYL in colon cancer, bladder cancer, and triple-negative breast cancer increases patient survival.^30^ In conclusion, ncRNAs play a vital role in human malignant tumors, and they can act as oncogenes or tumor suppressor genes to regulate the occurrence and progression of cancer. In addition, numerous ncRNAs can be detected in the blood or urine of cancer patients and can be employed as biomarkers for the early diagnosis of cancer.^31^ As a result, ncRNAs are an epigenetic modifier that cannot be overlooked in cancer early diagnosis, clinical targeted therapy, and prognosis evaluation.

To summarize, DNA methylation, histone modification, and ncRNA of epigenetic modification can not only function as biomarkers for early cancer diagnosis and prognosis but also control tumor suppressor gene or oncogene transcription and participate in a variety of biological processes involved in the occurrence and progression of cancer. In addition, non-cancerous cells in the tumor microenvironment (TME) are critical for the progression of malignant tumors.^32^ As a result, there is a lot of interest in the epigenetic modification of non-cancer cells in the TME. The epigenetic alterations of non-cancer cells in the TME may be the focus of future cancer-targeted therapy research.

## Epigenetic alteration of the TME

**Figure 2:**
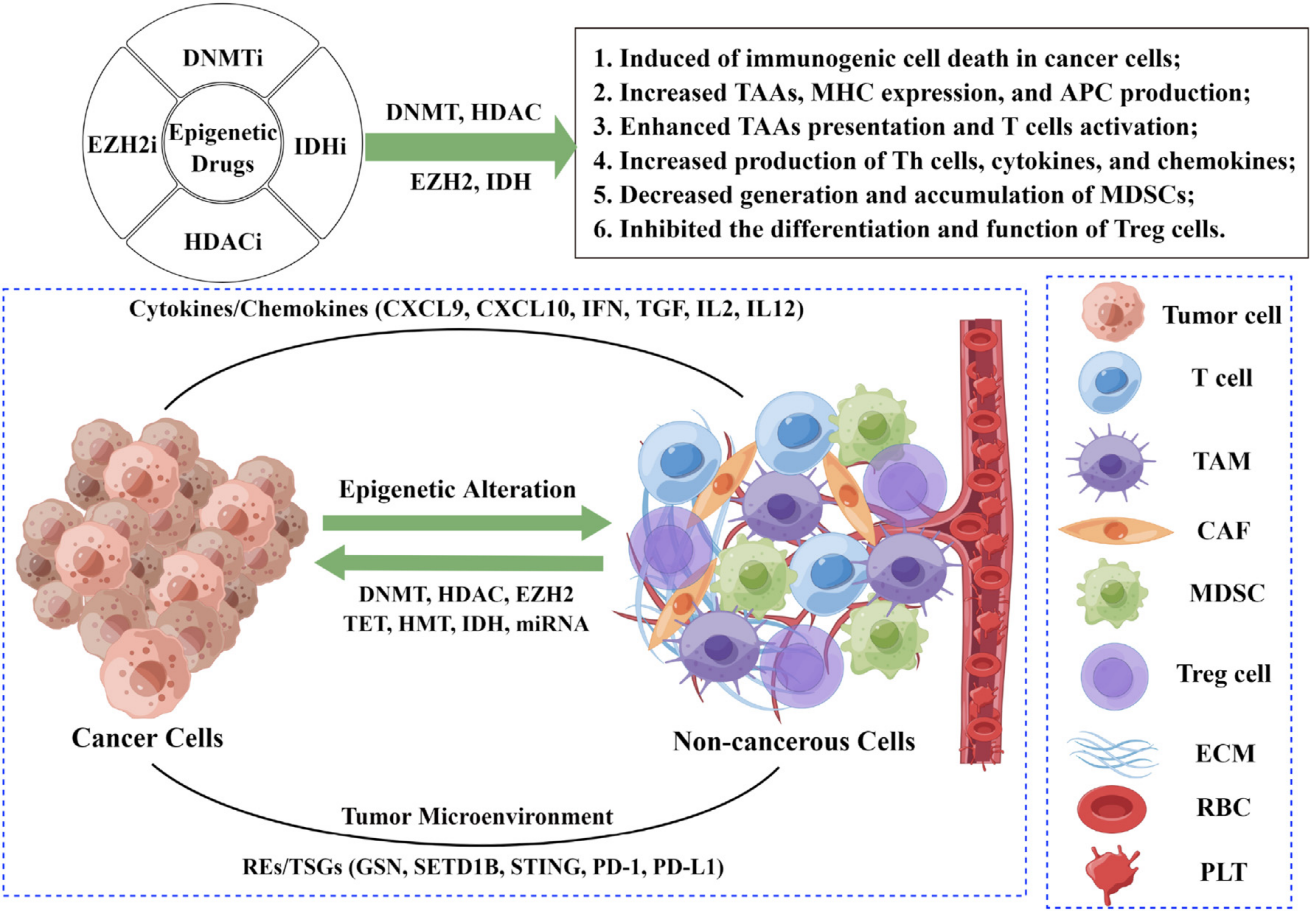
Potential roles of epigenetic alteration in cancer cells and non-cancerous cells in the tumor microenvironment. First, epigenetic drugs can induce immunogenic cell death in cancer cells and increase the expression of tumor-associated antigens (TAAs) and major histocompatibility complex (MHC) and the production of antigen-presenting cells (APCs), thereby enhancing cancer cell recognition and T cell activation by the presentation of cancer-associated antigens. Second, epigenetic drugs target immune cells in the tumor microenvironment, leading to reduced myeloid-derived suppressor cell (MDSC) production and accumulation as well as decreased regulatory T (Treg) cell differentiation and function. Third, during these processes, epigenetic drugs typically result in increased production of Th cells, cytokines, and chemokines, decreased extracellular matrix (ECM) secretion, and inhibition of tumor suppressor gene (TSG) expression. In conclusion, there are complex interactions in the tumor microenvironment between cancer cells and non-cancerous cells that induce epigenetic alterations in each other, and epigenetic modifications play a role in these interactions.

There is a crosstalk between cancer cells and non-cancer cells in the TME, which affect the occurrence and progression of cancer.^33^ On the one hand, TME is made up of a wide range of stromal cells, including cancer cells, fibroblasts, immune cells, endothelial cells, and extracellular matrix. Non-cancer cells in the TME can regulate the growth and metastasis of cancer cells by changing the TME state around the cancer cells. On the other hand, the lack of oxygen, acidity, and nutrients (glucose, lipids, and amino acids) in the TME also regulates the biological processes of cancer cells, including metabolic reprogramming, induction of proliferation, angiogenesis, inhibition of apoptosis, immune system suppression, and drug resistance.^34^ In addition, cancer cells also regulate the TME around them by suppressing the immune system and promoting cancer development. Recent research has revealed that epigenetic mechanisms are involved in these processes, as shown in Figure 2.^35^

## DNA methylation of non-cancer cells in the TME

As mentioned above, cancer cells usually exhibit overall hypomethylation and hypermethylation of tumor suppressor genes, whereas TME cells also exhibit DNA methylation changes that influence cancer progression. Cancer-specific changes in DNA methylation in cancer-associated fibroblasts (CAFs) have been reported. For example, CAFs in pancreatic cancer, ovarian cancer, and liver cancer exhibit global hypomethylation and gene-specific hypermethylation, whereas CAFs in prostate cancer do not show gene-specific hypomethylation and hypermethylation.^36^ Immune cells also showed changes in DNA methylation. Studies have shown that myeloid-derived suppressor cells contribute to immunosuppressive TME production, but exhibit overall hypomethylation.^37^ Specific DNMT3a is up-regulated in myeloid-derived suppressor cells, and the level of DNMT3a can shift immune function from a promoting to an inhibiting phenotype. Notably, the down-regulation of DNMT3a eliminates myeloid-derived suppressor cell-mediated inhibition of CD8^+^ T cell proliferation and interferon-γ production.^38^ In addition, Zou et al^39^ developed a DNA methylation signature signal based on CD8^+^ tumor-infiltrating lymphocytes that can be used to evaluate the immune response of CD8^+^ tumor-infiltrating lymphocytes in CRC, and the DNA methylation signal of CD8^+^ tumor-infiltrating lymphocytes may become a potential biomarker for CRC. In conclusion, DNA methylation of CAFs, myeloid-derived suppressor cells, and CD8^+^ tumor-infiltrating lymphocytes can be applied to the clinical diagnosis, treatment, and prognosis of cancer with significant clinical implications.

## Histone modifications of non-cancer cells in the TME

Non-cancer cells in the TME also exhibit histone modifications, similar to those seen in cancer cells. Epigenetic modifications have been shown to play an important role in driving non-cancer cell function in the TME. In CAFs of breast cancer, for example, HDAC6 is frequently up-regulated and promotes an immunosuppressive microenvironment. HDAC6 inhibits the recruitment of myeloid-derived suppressor cells and regulatory T cells, changes the phenotype of macrophages, and increases the activation of CD8^+^ T cells and CD4^+^ T cells *in vivo*. In addition, overexpression of HDAC6 in CAFs leads to the infiltration of myeloid-derived suppressor cells and regulatory T cells, which in turn promote breast cancer progression.^40^ It is known that CAFs enhance the secretion of abundant extracellular matrix to promote cancer progression and invasion, and secreted extracellular matrix protects cancer cells from immune checkpoint inhibitors or kinase inhibitors.^41^ It has been found that the HDAC inhibitor Scriptaid inhibits transforming growth factor-β-mediated conversion of endothelial cells and fibroblasts into CAFs, whereas HDAC overexpression induces extracellular matrix secretion.^42^

In addition to the histone acetylation pattern, the histone methylation pattern also occurs frequently in TME cells.^43^ H3K27me3 has been reported to play a crucial role in the progression of cancer in CAFs. CAFs secrete multiple stem cell ecotropic factors that confer invasiveness to cancer cells when H3K27me3 is lost. In addition, histone methylation also regulates bone marrow mesenchymal stem cells within the TME.^44^ It has been found that plasmacytoid dendritic cells produce type I interferon after HDAC inhibitor treatment, activating interferon gene transcription while promoting H3K27 acetylation. Conversely, plasmacytoid dendritic cell depletion reduces HDAC inhibitor-mediated type I interferon signaling in leukemic cells and attenuates its therapeutic effect.^45^ Thus, histone modifications in the TME in non-cancerous cells also have a significant impact on cancer cell progression.

## The role of non-coding RNA of non-cancer cells in the TME

As with DNA methylation and histone modifications, the role of ncRNA in the epigenetic modification of non-cancerous cells in the TME cannot be ignored. Recent studies have shown that lncRNAs are involved in tumor-stromal functional crosstalk and induce unique TME.^46^ The role of lncRNAs in the differentiation and function of immune cells (including T cells, dendritic cells, natural killer cells, tumor-associated macrophages, and myeloid-derived suppressor cells) in the TME has been reported to be critical.^47^ Additionally, N6-methyladenosine RNA (m6A RNA) modifications are the most abundant internal modifications in ncRNAs that can influence cancer progression and have great potential as tumor biomarkers and potential therapeutic targets.^48^ It has been found that cancer cells induce functional reprogramming of mesenchymal stem cells through the secretion of exosomes, thus promoting cancer progression. In addition to directly promoting their growth, these exosomes released by cancer cells also deliver molecular signals to fibroblasts, endothelial cells, and immune cells in the TME, indirectly enhancing their pro-tumorigenic effects and influencing the differentiation of non-cancerous cells in the TME.^49^ In addition, non-cancerous cells in the TME also release exosomal ncRNAs, which are involved in various biological processes in cancer. For instance, exosomal miRNA-144, miRNA-126, and miR-105 can regulate the metabolic reprogramming of non-cancerous cells in the TME through the Myc-AMPK-HIF1 pathway, thus affecting cancer proliferation, invasion, and migration.^50^ Similar to other ncRNAs, circRNAs can be transported from cancer cells to other cells in the microenvironment via exosomes, which are key messengers facilitating intracellular communication.^51^ For example, co-culture with highly metastatic LM3 (a hepatocellular carcinoma cell line) exosomes induced high migration and invasion of hepatocellular carcinoma cells that could be reversed upon knockdown of circPTGR1 expression. Additionally, circPTGR1, which targets miR449a and influences cancer cells' ability to migrate and invade, is released in exosomes from hepatocellular carcinoma cells. This disruption of TME homeostasis aids in the advancement of hepatocellular carcinoma.^50^ In conclusion, the differentiation and function of non-cancerous cells in the TME, metabolic reprogramming, exosomal secretion, and TME homeostasis are all impacted by ncRNA, which can control cancer growth and metastasis.

## Cancer cells and non-cancer cells in the TME induce epigenetic alterations in each other

Recent studies have shown that TME can affect epigenetic modifications in cancer cells, which can lead to cancer progression. For example, CAFs in ovarian cancer induce high expression of EZH2 in cancer cells, leading to increased cancer cell migration.^52^ Additionally, tumor-associated macrophages increase DNMT1 expression in gastric cancer cells, leading to the silencing of the tumor-suppressing gelsolin gene.^53^ In addition, both cancer cells and non-cancer cells in the TME release exosomes within the TME. Exosomes allow cancer and non-cancer cells to communicate with one another. Exosomal ncRNAs may regulate metabolic reprogramming, angiogenesis, epithelial–mesenchymal transition, and extracellular matrix remodeling to mediate cancer cell invasion and metastasis.^51^

Similarly, epigenetic dysregulation in cancer cells alters TME. For example, EZH2-mediated H3K27me3 and DNMT1-mediated DNA methylation in ovarian cancer reduce the levels of the chemokines CXCL9 and CXCL10 secreted by helper T cells (Th1), increasing CD8^+^ T cell infiltration, and inhibiting tumor growth.^54^ In addition, polycomb repressive complex 2-mediated epigenetic silencing of Th1-type chemokines inhibits effector T cell trafficking in colon cancer.^55^ Mediated methylation by DNMT1 and EZH2 has been reported to silence the miRNA-200 gene and promote the progression of gastric cancer and glioblastoma.^56^ Taken together, the above studies suggest that EZH2 and DNMT play a role in the formation of immunosuppressive TME, with complex interactions between cancer cells and non-cancerous cells in the TME that induce epigenetic changes in each other.

## Epigenetic drugs in cancer progression

As epigenetic modifications are clinically important in the development and progression of several cancers, there is a great deal of interest in the development of antitumor drugs that target epigenetic dysregulation.^57^ Currently, the FDA has approved ten epigenetic drugs for clinical application, including DNMT inhibitors azacitidine and decitabine, HDAC inhibitors vorinostat, romidepsin, belinostat, and panobinostat, isocitrate dehydrogenase (IDH) inhibitors ivosidenib and enasidenib, and EZH2 inhibitor tazemetostat (Table 1).^58^ Apart from these FDA-approved epigenetic drugs, other epigenetic drugs targeting DNMT, HDAC, ncRNA, bromodomain and extra-terminal (BET), disruptor of telomeric silencing 1-like (DOT1L), and LSD are in development. These include the DNMT inhibitors SGI-110, hydralazine, and HYB 101584, the HDAC inhibitors VPA, MGCD-0103, and 4SC-201, the ncRNA inhibitors MRG-106, MesomiR-1, CALAA-01, and ATU027, and the LSD inhibitors ORY-1001 and GSK2879552^4^ (Table 2).

**Table 1:** List of FDA-approved epigenetic drugs.

Targets	Drugs/inhibitors	Types of cancer/tumor	Year of approval
DNA methyltransferase (DNMT)	Azacitidine	Myelodysplastic syndromes/myeloproliferative neoplasm overlap syndromes, acute myeloid leukemia, chronic myelomonocytic leukemia	2004
	Decitabine		2006
Histone deacetylase (HDAC)	Vorinostat	Different subtypes of T-cell lymphoma	2006
	Romidepsin		2009
	Belinostat		2014
	Panobinostat		2015
	Chidamide		2015
Isocitrate dehydrogenase 1 (IDK1)	Ivosidenib	Acute myeloid leukemia	2017
Isocitrate dehydrogenase 2 (IDH2)	Enasidenib		2018
Enhancer of zeste homolog 2 (EZH2)	Tazemetostat	Epithelioid sarcoma, follicular lymphoma	2020

**Table 2:** List of some epigenetic drugs in clinical trials.

Targets	Drugs/inhibitors	Phase	Types of cancer/tumor
DNA methyltransferase (DNMT)	Guadecitabine (SGI-110)	Phase I	Cholangiocarcinoma, malignant melanoma, non-small cell lung cancer, pancreatic cancer, mesothelioma
		Phase II	Acute myeloid leukemia, chronic myelomonocytic leukemia, bladder cancer; myeloproliferative disorders, ovarian cancer
	Hydralazine (apresoline)	Phase I	Solid tumors
		Phase II	Cutaneous T-cell lymphoma, hepatocellular carcinoma
	MG98 (HYB 101584)	Phase II	Metastatic renal carcinoma
Histone deacetylase (HDAC)	VPA (valproic acid)	Phase II	Glioma, metastatic colorectal cancer
	Mocetinostat (MGCD-0103)	Phase I	Metastatic leiomyosarcoma, pancreatic cancer, melanoma
		Phase II	Solid tumors;
	Resminostat (4SC-201)	Phase I	Biliary cancer; solid tumors, pancreatic cancer
		Phase II	Hepatocellular carcinoma, non-small cell lung cancer, Hodgkin lymphoma, biliary cancer
Non-coding RNA	MRG-106	Phase I	Cutaneous T-cell lymphoma, diffuse large B-cell lymphoma, adult T-cell leukemia/lymphoma, chronic lymphocytic leukemia
	MesomiR-1	Phase I	Malignant pleural mesothelioma, non-small cell lung cancer
	CALAA-01	Phase I	Solid tumors
	ATU027	Phase I	Solid tumors
		Phase II	Pancreatic cancer
Bromodomain and extra-terminal (BET)	Molibresib (I-BET762)	Phase I/II	NUT carcinoma, solid tumors
Lysine-specific demethylase (LSD)	Iadademstat (ORY-1001)	Phase I/II	Acute myeloid leukemia
	GSK2879552	Phase I	Small cell lung cancer, acute myeloid leukemia
Disruptor of telomeric silencing 1-like (DOT1L)	Pinometostat (EPZ5676)	Phase I/II	Acute myeloid leukemia

## DNA methylation drugs

Epigenetic dysregulation has been linked to abnormal DNA hypermethylation. Measurable residual disease status after clinical intervention predicts early relapse and prognosis.^59^ When compared to the placebo group, the oral azacytidine group promoted the conversion of measurable residual disease-positive to -negative status and prolonged the duration of measurable residual disease-negative status in AML patients. Maintenance treatment with azacitidine significantly prolonged the overall survival and relapse-free survival of patients.^60^ According to the reports, the DNMT1 inhibitor decitabine activates the lncRNA PHACTR2-AS1 (PAS1) for recruitment of the histone methyltransferase SUV39H1 to cause H3K9 methylation of PH20, which results in its silencing.^61^ Recent studies have shown that DNMT inhibitors can act as sensitizers for antitumor drugs. By way of example, high expression of this cancer-testis antigen mRNA increases major histocompatibility complex class I antigen presentation in glioblastoma cells, enhances cancer-testis antigen-specific T cell activation, and potentiates activated T cell-mediated cytotoxicity of glioblastoma.^62^ Hypermethylation of oncogene promoters is known to silence them, thereby promoting cancer progression. It has been reported that neuronal pentraxin 2 expression is significantly reduced in prostate cancer tissues and cancer cell lines, which may be due to the high methylation of the neuronal pentraxin 2 promoter. Furthermore, decitabine inhibited the proliferation-promoting effect of neuronal pentraxin 2 mediated via its methylation in prostate cancer cells.^63^ As previously stated, DNA methylation drugs can modulate promoter methylation, lncRNA, and histone methylation, leading to epigenetic modifications of oncogenes and sensitizing the antitumor effects of antitumor drugs and the cytotoxic effects of immune cells.

## Histone modification drugs

In recent years, researchers have conducted extensive research into the use of HDAC inhibitors in various types of T-cell lymphoma. For example, romidepsin and mechlorethamine inhibit the proliferation and promote apoptosis of cutaneous T-cell lymphoma cells via the Janus kinase/signal transducer and activator of transcription pathway. Notably, this signaling pathway may be involved in antagonizing the resistance of romidepsin to cutaneous T-cell lymphoma.^64^ Clinical trials of the HDAC inhibitor tucidinostat in relapsed or refractory peripheral T-cell lymphoma and angioimmunoblastic T-cell lymphoma have shown that the overall response rate is significantly improved. This was, of course, accompanied by an increase in the number of cases in complete remission and partial remission.^65^ PCI-24781, a novel glioblastoma signature-specific HDAC inhibitor, has been shown to significantly down-regulate O6-methylguanine-DNA-methyltransferase expression, increase DNA double-strand breaks, and lead to increased apoptosis in glioblastoma cell lines. In addition, PCI-24781 reduced tumor load in a genetically engineered mouse model of glioblastoma.^66^ Previous studies have demonstrated that IDH1/2 mutant cancer cells produce the tumor metabolite 2-hydroxyglutarate (2-HG) and that 2-HG-induced histone hypermethylation results in homology-directed repair defects. In addition, vorinostat causes increased DNA double-strand breaks and functional homology-directed repair deficiency by down-regulating the expression of the DNA repair factors breast cancer 1 (BRCA1) and Rad51, resulting in increased cell death in IDH1 mutant cancer cells.^67^ The combined intervention of romidepsin and tamoxifen in pancreatic cancer cells was reported to induce reactive oxygen species production and mitochondrial lipid peroxidation, which further led to apoptosis. In addition, the expression of forkhead box protein M1 was significantly reduced in romidepsin monotherapy-treated pancreatic cancer cells. As a result, forkhead box protein M1 has the potential to be used as a clinical therapeutic target as well as a prognostic marker for pancreatic cancer.^68^ In conclusion, histone-modifying drugs can inhibit cancer cell proliferation, promote apoptosis, and increase overall response rate, partial remission, and complete remission in patients by regulating the Janus kinase/signal transducer and activator of transcription pathway, DNA damage repair, and mitochondrial metabolism.

## Drugs that target non-coding RNAs

As previously stated, the FDA-approved epigenetic modification drugs for clinical application are primarily DNMT inhibitors and HDAC inhibitors. Although drugs that regulate ncRNAs are still in phase I and II of clinical trials, they still hold great promise for clinical application. For example, miR-155 is a miRNA linked to poor prognosis in lymphoma and leukemia. MRG-106 inhibits miR-155 expression and regulates multiple survival pathways (including Janus kinase/signal transducer and activator of transcription, mitogen-activated protein kinase/extraneous signal-regulated kinase, and phosphatidylinositol 3-kinase/protein kinase B) to reduce cell proliferation and survival in cutaneous T-cell lymphomas.^69^ Additionally, Atu027, a siRNA-lipid complex targeting protein kinase N3, significantly inhibited tumor growth and lymph node metastasis in *situ* mouse models of prostate and pancreatic cancer.^70^ CALAA-01 and MesomiR-1, two other ncRNA regulatory drugs, have also been reported to be in phase I clinical trials in solid tumors, malignant pleural mesothelioma, and non-small cell lung cancer.^71^

In addition to DNA methylation drugs, histone modification drugs, and ncRNA modulating drugs, epigenetic drugs targeting BET, LSD, and DOT1L targets are also in clinical phase I and II trials.^72^ Taken together, epigenetic drugs have obvious prospects for clinical application in terms of enhancing the antitumor effects of other anticancer drugs, changing the status of TME (inhibition or activation), and reversing drug resistance to some extent, but there are also issues of heterogeneity of epigenetic drugs and heterogeneity of cancer cells, as well as adverse effects.^73^ As a result, combining epigenetic drugs may open up new avenues for future clinical cancer treatment.

## Epigenetic combination therapies may be novel strategies for cancer treatment

**Figure 3:**
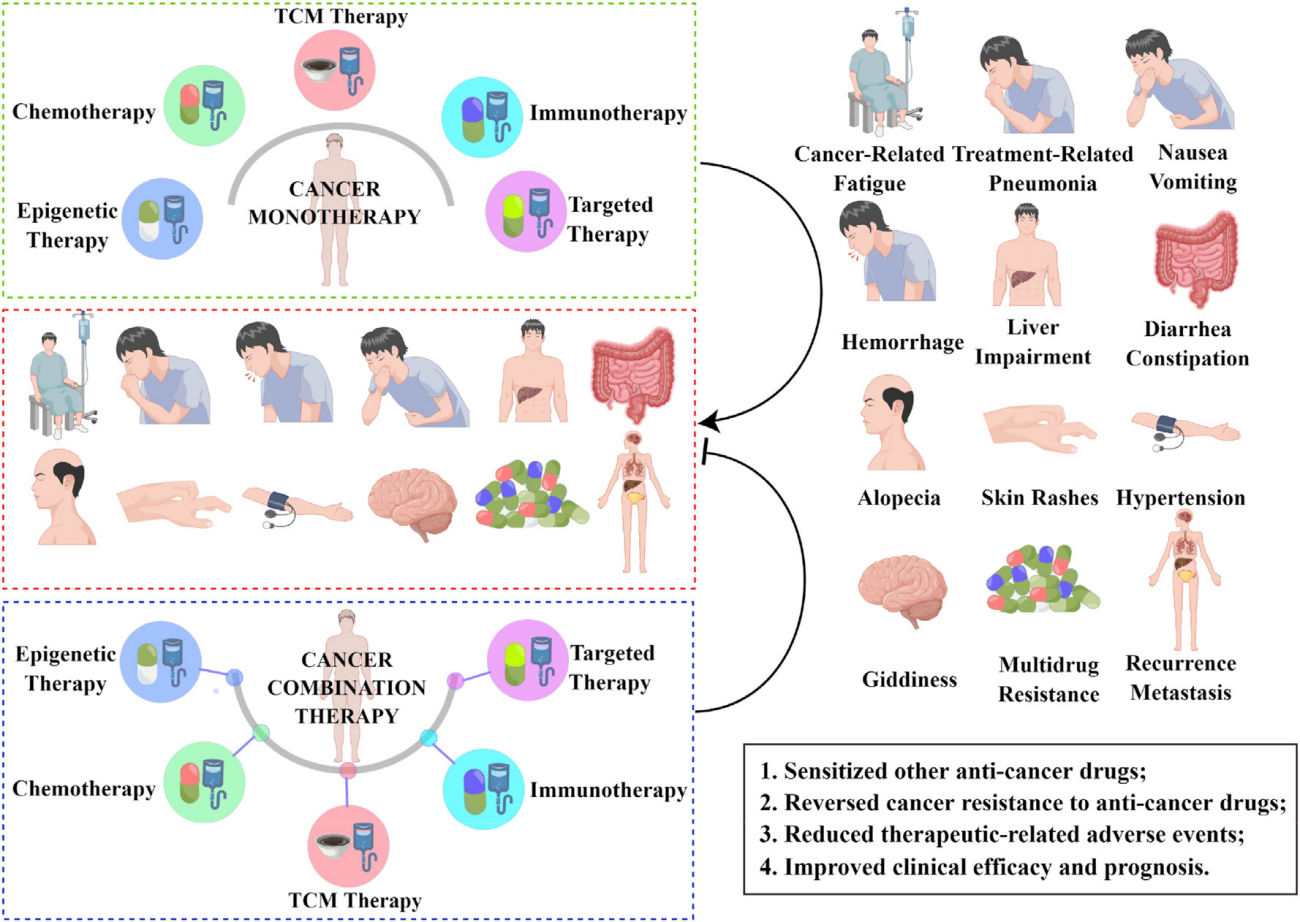
Potential benefits of epigenetic combination therapy in the clinical management of cancer. Cancer monotherapies are associated with a variety of adverse events in the clinical management of patients, including cancer-related fatigue, treatment-related pneumonia, nausea and vomiting, hemorrhage, liver impairment, diarrhea and constipation, alopecia, skin rashes, hypertension, and giddiness. In addition, cancer monotherapies are mostly prone to multidrug resistance, recurrence, and metastasis, all of which have a negative impact on the quality of life and clinical outcomes of patients. Epigenetic drugs have shown great potential in cancer treatment. Epigenetic combination therapy not only enhances antitumor effects and reduces resistance to other anticancer drugs but also lowers the incidence of treatment-related adverse events. In addition, epigenetic combination therapy may improve the clinical outcome and prognosis of patients to some extent. In conclusion, epigenetic combination therapy may be a promising strategy for cancer treatment.

As a result of cancer's susceptibility to recurrence, metastasis, and malignant progression, single therapies or drugs often fail to achieve the desired efficacy. While combination strategies of targeted agents, immunologic agents, and chemotherapeutic agents have been included in clinical guidelines for cancer treatment, some cancer patients have limited clinical benefit, especially those with refractory or recurrence-prone cancers, as shown in Figure 3.^74^ Consequently, active research and the development of new therapeutic tools and drugs are the current research priorities in cancer clinical treatment. Epigenetic modifications have been a hot topic in cancer clinical treatment research in recent years, and the research and development of drugs targeting epigenetic modifications is in full swing. Simultaneously, the combination of epigenetic drugs and their combinations with other anti-cancer drugs are in phase I, II, and III clinical trials, and epigenetic drug combination strategies may be a new perspective for cancer clinical treatment.^57^^,^^75^

## Combination therapy between epigenetic drugs

In recent years, the role of epigenetic modifications in cancer progression has been increasingly studied, and combinations of epigenetic agents are in preclinical or clinical research stages, as shown in Table 3. In a reported clinical study of 146 patients with IDH1 mutated AML, the combination group of ivosidenib and azacitidine significantly prolonged the median overall survival and reduced the incidence of common adverse events.,^76^ which is generally consistent with the findings of the AML-295 AGILE phase 3 clinical trial.^77^ Another study of AML with mutant IDH2 (AG221-AML-005) found that the overall remission rate in patients in the combination group of enasidenib and azacitidine was 4.9 times higher than in the azacitidine monotherapy group and was accompanied by treatment-related adverse events including thrombocytopenia, neutropenia, anemia, and febrile neutropenia.^78^ Multiple clinical studies have shown that the combination of DNMT inhibitors and HDAC inhibitors is safe and effective. For example, the combination of azacitidine and butyrate significantly reduced cancer stem cell abundance as well as the expression levels of Rad51AP1 and SPC25, proteins associated with DNA damage repair, further affecting the progression and prognosis of breast cancer.^79^ As a result, the active development of multifunctional inhibitors targeting DNMT and HDAC is of great significance for cancer clinical treatment. C02S is an epigenetic drug that specifically targets DNMT1, DNMT3a, DNMT3b, and HDAC1. C02S reverses mutational methylation and acetylation in cancer cells, up-regulates the expression of tumor suppressor proteins, and regulates a variety of biological processes, including the induction of apoptosis and cell cycle arrest, inhibition of angiogenesis, blocking migration and invasion, and ultimately inhibiting cancer progression.^80^ As a consequence, epigenetic drug combinations that prolong median patient survival and improve overall response rate, complete remission, and partial remission are a boon for patients with recurrent or refractory cancer who are ineligible for chemotherapy.

**Table 3:** List of clinical trials of epigenetic combination therapies in cancer treatment.

Epigenetic therapy	Other therapy	Phase	Cancer type	Primary outcome measures	Clinical trial
Azacitidine	Ivosidenib	Phase III	Mutant-IDH1 acute myeloid leukemia	OS EFS	NCT03173248
Azacitidine	Enasidenib	Phase I/II	Mutant-IDH2 acute myeloid leukaemia	OS ORR	NCT02677922 NCT03683433
Azacitidine Decitabine	Venetoclax	Phase I	Acute myeloid leukaemia	OS MTD ORR	NCT02203773 NCT02993523
Azacitidine	Gilteritinib	Phase III	Acute myeloid leukemia	OS	NCT02752035
Azacitidine	Pembrolizumab	Phase II	Metastatic colorectal cancer Myelodysplastic syndrome	OS ORR PFS	NCT02260440 NCT03094637
Guadecitabine	Carboplatin	Phase II	Ovarian Cancer	OS PFS	NCT01696032
Azacitidine	Avelumab	Phase I/II	Acute myeloid leukemia	OS ORR	NCT02953561
Azacitidine	Nivolumab	Phase II	Acute myeloid leukemia	ORR	NCT02397720
Azacitidine	Gemtuzumab	Phase I/II	Acute myeloid leukemia	MTD	NCT00766116
Azacitidine	R–CHOP	Phase I	Diffuse large B-cell lymphoma	MTD	NCT02343536
Decitabine vorinostat	FLAG	Phase I	Acute myeloid leukemia	OS MTD ORR	NCT02412475
Chidamide Decitabine	CDCAG	Phase I/II	Acute myeloid leukemia	OS ORR	NCT02886559

Note: OS, overall survival; EFS, event-free survival; ORR, objective remission rate; MTD, maximum tolerated dose; PFS, progression-free survival; R–CHOP, rituximab, cyclophosphamide, doxorubicin, vincristine, and prednisone; FLAG, fludarabine, cytarabine, and granulocyte colony-stimulating factor; CDCAG, aclarubicin, cytarabine, and granulocyte colony-stimulating factor.

## Epigenetic drugs combined with chemotherapy

Chemical drugs are one of the most common clinical treatments for cancer, but cancer cell resistance to chemical drugs has a serious impact on patient efficacy and quality of life. Several studies have found that the development of chemoresistance is linked to epigenetic modifications, such as hypermethylation of DNA, high expression of HDAC, and suppression or high expression of miRNA.^81^ Azacitidine has been shown to be effective in treating recurrent ovarian cancer after chemotherapy and treatment-related AML with a good clinical response.^82^ Platinum chemoresistance is known to cause the recurrence of high-grade plasma cell ovarian cancer. Studies have shown that the clinical efficacy of the azacitidine and carboplatin sequential treatment group was superior to that of carboplatin monotherapy, which may be associated with enhanced demethylation and enhanced immune response, thereby slowing the growth of platinum-resistant ovarian cancer cells.^83^ Furthermore, triple-negative breast cancers were highly sensitive to decitabine, which increased the apoptosis regulator NOXA. Decitabine increased cisplatin's NOXA-mediated pro-apoptotic effect on breast cancer cell lines, demonstrating decitabine's good clinical potential in combination with cisplatin for the treatment of triple-negative breast cancers.^84^ It has been shown that mitochondrial DNA depletion and low mitochondrial membrane potential can induce DNA methylation via DNMT and that zebularine inhibits epithelial–mesenchymal transition and improves chemotherapy sensitivity in mitochondrial DNA-depleted esophageal cancer cells. As a result, therapeutic strategies that increase the copy number of mitochondrial DNA and DNMT inhibitors may be useful in preventing epithelial–mesenchymal transition and chemotherapy resistance.^85^

## Epigenetic drugs combined with targeted therapy

Currently, researchers are conducting in-depth research on the exploration and development of drugs targeting cancer therapy, and an increasing number of cancer targets and targeted drugs are being introduced. Even though the targeted drugs have demonstrated clinical efficacy, there are still problems such as drug resistance and adverse effects. Enzalutamide is a drug that has been approved by the FDA for the treatment of patients with advanced prostate cancer. Unfortunately, after a short positive response period to enzalutamide, tumors will develop resistance to the drug. Enzalutamide has been shown in recent studies to increase the expression of DNMT3a and DNMT3b in prostate cancer cells. In addition, the knockdown of DNMT3b or decitabine sensitizes cancer cells to enzalutamide. Thus, enzalutamide resistance may be linked to abnormal DNA methylation.^86^ It was reported that poly ADP-ribose polymerase inhibitors had been approved for the treatment of prostate cancer with BRCA mutations, but the clinical efficacy was unsatisfactory. One study found that veliparib in combination with vorinostat inhibited the proliferation and growth of prostate cancer by targeting the UHRF1/BRCA1 protein complex, which promotes DNA damage and inhibits DNA damage repair pathways.^87^ Reportedly, after a brief treatment with gilteritinib alone in patients with mixed phenotype acute leukemia who relapsed after chemo-induction with limited clinical efficacy, patients were started on a combination of gilteritinib and azacitidine, which eventually achieved and maintained complete remission with incomplete blood cell count recovery for 8 months.^88^ As shown in Table 3, epigenetic drugs can partially reverse cancer cell resistance to chemotherapeutic and targeted drugs, enhance the antitumor effects of chemotherapeutic and targeted drugs, and improve overall response rate in cancer patients. In conclusion, epigenetic drugs combined with chemotherapy and targeted therapy can help prevent the development of drug resistance and prolong the survival of patients.

## Epigenetic drugs combined with immunotherapy

Inhibitors targeting immune checkpoints have been widely used in the treatment of a variety of clinical cancers, exerting their antitumor effects by modulating the function of immune cells but failing to respond to some refractory or recurrent cancers. The two most common immune checkpoints in cancer research are programmed cell death-1 and programmed cell death ligand 1. Their expression has been reported to be up-regulated in myelodysplastic syndromes, and hypomethylating agent treatment resulted in a further increase in their expression.^89^ Additionally, patients with chemotherapy-resistant metastatic colorectal cancer did not respond to pembrolizumab monotherapy. When azacitidine and pembrolizumab were combined for chemotherapy-refractory metastatic colorectal patients, the levels of genomic DNA methylation and promoter region methylation were significantly downregulated, accompanied by up-regulation of immune-related genes and CD8^+^ tumor infiltrating lymphocyte density.^90^ Clinical treatment options for patients with relapsed or refractory AML are relatively limited, and avelumab, an anti-PD-L1 immune checkpoint inhibitor, is approved for use in a variety of solid tumors.^91^ Epigenetic inhibitors induce a wide range of changes in gene expression in cancer cells, and combining them with chimeric antigen receptor T cell therapy may contribute to cancer treatment. For example, decitabine increases the cytotoxicity of chimeric antigen receptor T cells against uroepithelial cancer cell lines. Gene expression analysis showed that decitabine differentially induced regulators of cell survival and apoptosis into an apoptosis-sensitive state associated with increased chimeric antigen receptor T cell killing.^92^ It should be noted that “cold” TME influences the clinical response to immune checkpoint inhibitors in patients with colorectal cancer who have microsatellite stability. Decitabine has been shown to promote the transition from “cold” to “hot” TME by increasing the expression of the tumor-associated antigen NY-ESO-1. Meanwhile, NY-ESO-1 may be an ideal target for antigen-specific T cell receptor-engineered T cells.^93^ From the above, the combination of epigenetic drugs and immunotherapy may be a promising novel strategy for cancer treatment.

## Epigenetic drugs combined with TCM

TCM exerting its anti-tumor effects through multi-channel, multi-target, and multi-component characteristics has been widely employed in the treatment of cancer.^34^^,^^94^ According to the report, a reduction in deoxycytidine kinase, the activating enzyme of decitabine, was found in acquired, decitabine-resistant colorectal cancer HCT116 cells. Notably, the combination of curcumin and azacitidine reversed the resistance of HCT116 cells to decitabine by altering the expression of deoxycytidine kinase.^95^ Recent research has linked HSP72 overexpression to resistance to HDAC drugs in Hut78 cells. Furthermore, modest dosages of quercetin inhibited Hut78 cell growth by decreasing HSP72 expression, increasing HDAC activity, and improving vorinostat-induced inhibition. Quercetin also significantly enhanced vorinostat-induced apoptosis and loss of mitochondrial membrane potential.^96^ DNMT1, DNMT3b, HDAC1, and HDAC3 have been reported to be highly expressed in choriocarcinoma cancer stem-like cells. Curcumol suppresses their stemness by inhibiting the activities of DNMTs and HDACs. Curcumol was found to be more effective at removing their stemness of than a combination of DNMT and HDAC inhibitors. Therefore, combining curcumol with epigenetic inhibitors may be a novel strategy for the targeted treatment of choriocarcinoma cancer stem-like cells.^97^ Berberine increases PTEN expression while decreasing phosphatidylinositol 3-kinase/protein kinase B/mTOR pathway expression, inhibiting colorectal cancer SW480 cell proliferation, and inducing apoptosis, mitochondrial membrane potential depolarization, and autophagy. Berberine has synergistic anti-colorectal cancer effects when combined with an HDAC inhibitor.^98^ Besides, baicalein, magnoliol, and emodin inhibit proliferation and promote apoptosis in cutaneous T-cell lymphoma, AML, hepatocellular carcinoma, and colorectal cancer by regulating the expression of HDAC1 and HDAC8. Thus, the clinical and basic research of TCM in combination with epigenetic drugs may be an important direction for future targeted cancer therapy.

## Multiple anti-cancer drug combinations

The issues of limited efficacy, heterogeneity, and resistance of clinical therapeutic agents for cancer, particularly for relapsed or refractory cancers, remain a significant challenge to inhibiting cancer progression.^99^ It is well known that resistance to standard immunochemotherapy in diffuse large B-cell lymphoma severely affects patients' quality of life and prognosis, while aberrant DNA methylation may lead to chemoresistance. It has been shown that oral azacitidine in combination with rituximab, cyclophosphamide, doxorubicin, vincristine, and prednisolone in previously untreated patients with intermediate-to-high risk diffuse large B-cell lymphoma had an overall response rate of 94.9%, with 46 of 52 patients achieving complete remission, as described in Table 3.^100^ Currently, clinical treatment options for relapsed or refractory pediatric AML patients remain limited. Decitabine and vorinostat combined with fludarabine, cytarabine, and granulocyte colony-stimulating factor were reported to treat 35 pediatric patients with relapsed or refractory AML with an overall response rate of 54%, as described in Table 3.^101^ In addition, patients with relapsed or refractory AML who received the combination of chidamide, decitabine, cytarabine, atramycin, and granulocyte colony-stimulating factor had an overall response rate of 46.2%, with 24 achieving complete remission and 19 reaching complete remission with incomplete blood count recovery (Table 3).^102^ In conclusion, the combination strategy of multiple antitumor agents is a preferred option for relapsed or refractory cancers to antagonize the resistance of some drugs and enhance the oncogenic effects of other anticancer drugs, thereby increasing patient survival and improving prognosis.

## Conclusion and prospects

In recent years, with the application of technologies such as high-throughput single-cell sequencing and epigenomics, more and more epigenetic modifications have been discovered in cancer progression, and active research and development of epigenetic drugs have important clinical significance. There is evidence that epigenetic alterations play a role in cancer resistance to the aforementioned anticancer drugs.^103^ Additionally, epigenetic drug combination strategies have shown significant benefits for patients with recurrent or refractory cancers that do not respond to chemotherapy, targeted or immune cell-based therapies, or standard first-line treatment regimens.^90^^,^^91^^,^^101^^,^^102^

This review summarizes the role of epigenetic modifications in the TME and cancer progression, the antagonism of cancer progression by epigenetic drugs, and the clinical application and advantages of epigenetic drug combination strategies in cancer development and progression. It also explains the great potential of epigenetic drugs as antagonists of cancer progression and epigenetic drug combination strategies against tumors. Epigenetic drugs can be used as sensitizers for other antitumor drugs as well as antagonists of anticancer drug resistance. In addition, epigenetic drugs can regulate the expression of DMNTs, HDACs, ncRNAs, oncogenes, and tumor suppressor genes in cancer cells, as well as epigenetic alterations in non-cancerous cells in the TME, thereby regulating cancer development and progression. Most importantly, for recurrent or refractory cancers, the combination strategy of epigenetic drugs combined with epigenetic drugs, chemical drugs, targeted drugs, immune drugs, and TCM shows significant advantages and clinical potential. In summary, epigenetic drugs and their combination strategies have the potential to be effective cancer treatment strategies.

The development of both molecular mechanisms and new drug targets for epigenetic modifications to regulate cancer is well underway. However, epigenetic modifications also face unique challenges. For example, clinical trials of epigenetic drugs and their combination strategies have been linked to adverse events, which have a negative impact on patient adherence and quality of life.

It is well known that an important area for future research in cancer epigenetics is the epigenetic heterogeneity of cancer. For example, in the drug target identification or validation phase, we have not yet fully clarified the epigenetic landscape of all cell types (normal cells and cancer cells) from structural and functional level perspectives, and the heterogeneity of cancer cells needs to be considered in the development of epigenetic drugs. In addition, the heterogeneity of epigenetic drugs is also a source of concern. For example, FDA-approved epigenetic drugs for clinical use are effective in only some cancers. Notably, future epigenetic modification research will focus on the continuous discovery of new epigenetic agents and biomarkers. Therefore, actively developing drugs that act on multiple target epigenetic agents simultaneously is an important step to overcoming epigenetic heterogeneity. In conclusion, there are reasons to believe that with the application of high-throughput sequencing and genomics technologies, epigenetics-related research on cancer will continue to advance, and epigenetic drugs will be a rising jewel in the field of cancer clinical treatment.

## Author contributions

JYM, LT, and SHL contributed to the direction and guidance of this review. JYM, LT, and SHL contributed equally to this study and shared the corresponding authorship. DW and YZ drafted the manuscript and revised the manuscript. DW and YZ contributed equally to this work and shared the first authorship. QBL, YL, and WL prepared the figures. AZ and JXX made the tables of the manuscript. All authors read and approved the final manuscript.

## Conflict of interests

All authors have significantly contributed, and all authors agree with the content of the manuscript. All authors declare no conflict of interests.

## Funding

This work was supported by the 10.13039/501100001809National Natural Science Foundation of China (No. 82274396, 82204961).
